# Supplementary material for: An essential role for MEF2C in the cortical response to loss of sleep in mice
Source: eLife. 2020 Aug 27;9:e58331. doi: 10.7554/eLife.58331 (PMC7490011; doi:10.7554/eLife.58331)
Supplement: Supplementary file 7. — P2/P1 was obtained at three different interpulse intervals (20, 50 and 100 ms), obtained in anterior cingulate cortex excitatory neurons of Mef2cf/f and Mef2c-cKOCamk2a-Cre mice exposed to three different sleep/wake experimental conditions: control sleep 6 hr (CS), sleep deprivation 6 hr (SD) and sleep deprivation 4 hr followed by recovery sleep 2 hr (RS). [file elife-58331-supp7.docx]

**Supplementary Table 7. Paired pulse ratio of evoked EPSCs at three different interpulse intervals (20, 50 and 100 ms), obtained in anterior cingulate cortex excitatory neurons of Mef2c^f/f^ and Mef2c^CKO^ mice exposed to three different sleep/wake experimental conditions: control sleep 6h (CS), sleep deprivation 6h (SD) and sleep deprivation 4h followed by recovery sleep 2h (RS).**

| **P2/P1 ratio** | | **Mef2c^f/f^** | | | | | | | | **Mef2c^CKO^** | | | | | | | | |
| --- | --- | --- | --- | --- | --- | --- | --- | --- | --- | --- | --- | --- | --- | --- | --- | --- | --- | --- |
|  | | *Fig.5 B* | | | | | | | | *Fig.5 D* | | | | | | | | |
| *From N cell experimental values* | | N | Mean ± SEM | ANOVA F, (DFn, DFd) | ANOVA, Adjusted P value | | | | | N | Mean ± SEM | ANOVA F, (DFn, DFd) | | ANOVA, Adjusted P value | | | | |
|  |  |  |  |  | CS | SD | | RS | |  |  |  |  | CS | SD | | RS | |
| 20 ms interpuls time, CS | | 8 | 0.72±0.07 | 3.27  (2,20) | N/A | 0.42 | | 0.047 | | 9 | 0.88±0.09 | 1.54  (2,20) | | N/A | 0.22 | | 0.88 | |
| 20 ms interpuls time, SD | | 7 | 0.87±0.07 |  | 0.42 | N/A | | 0.48 | | 9 | 1.04±0.04 |  |  | 0.22 | N/A | | 0.60 | |
| 20 ms interpuls time, RS | | 8 | 1.00±0.09 |  | 0.047 | 0.48 | | N/A | | 5 | 0.93±0.06 |  |  | 0.88 | 0.60 | | N/A | |
| 50 ms interpuls time, CS | | 8 | 0.77±0.04 | 4.71  (2,21) | N/A | 0.16 | | 0.017 | | 9 | 0.86±0.06 | 1.42  (2,21) | | N/A | 0.24 | | 0.65 | |
| 50 ms interpuls time, SD | | 8 | 0.93±0.07 |  | 0.16 | N/A | | 0.50 | | 9 | 1.02±0.07 |  |  | 0.24 | N/A | | 0.82 | |
| 50 ms interpuls time, RS | | 8 | 1.03±0.07 |  | 0.017 | 0.50 | | N/A | | 6 | 0.96±0.09 |  |  | 0.65 | 0.82 | | N/A | |
| 100 ms interpuls time, CS | | 10 | 0.75±0.04 | 1.86  (2,23) | N/A | 0.21 | | 0.31 | | 9 | 0.89±0.06 | 2.32  (2,22) | | N/A | 0.11 | | 0.34 | |
| 100 ms interpuls time, SD | | 8 | 0.89±0.06 |  | 0.21 | N/A | | 0.97 | | 9 | 1.06±0.05 |  |  | 0.11 | N/A | | 0.86 | |
| 100 ms interpuls time, RS | | 8 | 0.87±0.06 |  | 0.31 | 0.0.97 | | N/A | | 7 | 1.01±0.06 |  |  | 0.34 | 0.86 | | N/A | |
|  |  | *Fig.5 B* | | | | | | | | *Fig.5 D* | | | | | | | | |
| *From N cell experimental values* | | N | Median | 25% -tile | 75% -tile | | Min value | | Max value | N | Median | | 25% -tile | 75% -tile | | Min value | | Max value |
| 20 ms interpuls time, CS | | 8 | 0.71 | 0.62 | 0.86 | | 0.38 | | 1.06 | 9 | 0.95 | | 0.61 | 1.09 | | 0.46 | | 1.22 |
| 20 ms interpuls time, SD | | 7 | 0.93 | 0.73 | 0.99 | | 0.52 | | 1.02 | 9 | 1.06 | | 0.94 | 1.16 | | 0.83 | | 1.22 |
| 20 ms interpuls time, RS | | 8 | 1.06 | 0.77 | 1.19 | | 0.57 | | 1.31 | 5 | 0.89 | | 0.82 | 1.07 | | 0.79 | | 1.14 |
| 50 ms interpuls time, CS | | 8 | 0.81 | 0.66 | 0.86 | | 0.54 | | 0.89 | 9 | 0.89 | | 0.71 | 0.94 | | 0.66 | | 1.20 |
| 50 ms interpuls time, SD | | 8 | 0.96 | 0.75 | 1.11 | | 0.63 | | 1.14 | 9 | 1.00 | | 0.91 | 1.11 | | 0.71 | | 1.44 |
| 50 ms interpuls time, RS | | 8 | 1.01 | 0.89 | 1.17 | | 0.77 | | 1.34 | 6 | 0.98 | | 0.75 | 1.09 | | 0.70 | | 1.28 |
| 100 ms interpuls time, CS | | 10 | 0.72 | 0.65 | 0.89 | | 0.55 | | 0.97 | 9 | 0.91 | | 0.74 | 0.98 | | 0.67 | | 1.24 |
| 100 ms interpuls time, SD | | 8 | 0.92 | 0.73 | 1.06 | | 0.59 | | 1.07 | 9 | 1.06 | | 0.91 | 1.18 | | 0.85 | | 1.29 |
| 100 ms interpuls time, RS | | 8 | 0.89 | 0.73 | 0.98 | | 0.60 | | 1.18 | 7 | 1.01 | | 0.94 | 1.13 | | 0.75 | | 1.26 |
